# Antimicrobial Resistance of* Salmonella enterica* Serovars Typhi and Paratyphi Isolates from a General Hospital in Karawaci, Tangerang, Indonesia: A Five-Year Review

**DOI:** 10.1155/2017/6215136

**Published:** 2017-04-06

**Authors:** Nata Pratama Hardjo Lugito, Cucunawangsih

**Affiliations:** ^1^Department of Internal Medicine, Faculty of Medicine, Pelita Harapan University, Tangerang, Banten, Indonesia; ^2^Department of Microbiology, Faculty of Medicine, Pelita Harapan University, Tangerang, Banten, Indonesia

## Abstract

Typhoid and paratyphoid fever known as enteric fever pose important global public health problem, with 21.6 million cases and approximately 250,000 deaths annually. It is a prevalent disease in Indonesia, but data on the antimicrobial resistance pattern is limited. This study aim was to provide data on the antimicrobial resistance pattern of* S. *Typhi and* S. *Paratyphi bloodstream isolates in a general hospital in Karawaci, Tangerang, Banten, Indonesia, during the period of January 2011 to December 2015. Susceptibility against antimicrobials was detected according to the guidelines of the Clinical and Laboratory Standards Institute (CLSI). Out of a total of 168 isolates 55.4% were* S. *Typhi and 44.6%* S. *Paratyphi A. Most of the isolates, 92.9%, were from children aged 6–18 years and adult population. There was low resistance of* S. *Typhi against ampicillin, trimethoprim-sulfamethoxazole, ceftriaxone, ciprofloxacin, and levofloxacin, similar to previous studies in Indonesia. In the 2011–2015 period, resistance rates against most antimicrobials and MDR rate of* S. *Typhi and* S. *Paratyphi were low, emphasizing that there is a distinct epidemiological dynamic of the enteric fever in Indonesia.

## 1. Introduction

Enteric fever, both typhoid and paratyphoid fever are important global public health problems, with 21.6 million cases and approximately 250,000 deaths annually [1, 2]. Typhoid and paratyphoid fever are caused by* Salmonella enterica *serovars Typhi (*Salmonella* ser. Typhi) and Paratyphi, respectively. It is estimated that more than 90% of typhoid fever cases were reported in South and Southeast Asian countries [1].

In many parts of the world, the changing modes of presentation and the development of multidrug resistance have made enteric fever increasingly difficult to diagnose and treat. Multidrug resistant (MDR)* S. *Typhi is defined as* S*. Typhi strains which are resistant to all the three first-line recommended drugs for treatment,* that is*, chloramphenicol, ampicillin, and trimethoprim-sulfamethoxazole [3]. There had been reports on resistance of* Salmonella* species against antimicrobial used, beginning with the report of chloramphenicol resistance in 1972 to the report of multidrug resistant strains [4, 5]. The first MDR strains were discovered in Southeast Asia in the late 1980s and have since spread throughout the region [6]. The use of first-line antimicrobial for the treatment of enteric fever such as ampicillin, chloramphenicol, and trimethoprim-sulfamethoxazole had been recommended to be replaced with quinolone or 3rd generation cephalosporins [7]. In Indonesia, MDR* S.* Typhi was reported in Surabaya, in 2009 [8]. A multicentric study conducted across five Asian countries endemic for typhoid including Indonesia reported that the prevalence of MDR* S*. Typhi ranged from 7% to 65% [9].

Enteric fever is a prevalent disease in Indonesia, but data on the antimicrobial resistance pattern is limited. The aim of this study was to provide data on the antimicrobial resistance pattern of* S*. Typhi and* S. *Paratyphi isolates from patients admitted to a general hospital in Karawaci, Tangerang, Banten, Indonesia during the period of 2011–2015.

## 2. Material and Method

### 2.1. Study Setting

This retrospective descriptive study was conducted in Siloam Hospital in Karawaci, Tangerang, Banten, Indonesia. The hospital is a private teaching hospital with 274 beds, affiliated to Faculty of Medicine, Pelita Harapan University. The samples in this study were all bloodstream isolates of* S. *Typhi and* S. *Paratyphi collected at the Microbiology Laboratory of the hospital.

### 2.2. Specimen, Culture, and Identification

Venous blood samples were collected from patients admitted to hospital from January 2011 to December 2015. Blood was inoculated into enriched soybean-casein digest broth with resins in BACTEC aerobic plus/F (Becton-Dickinson, New Jersey, USA) bottles. For patients with body weight less than 12.8 kg of weight, BACTEC Peds Plus/F bottles were used. When there was bacterial growth indicated by the BACTEC machine, blood culture bottles were subcultured onto a MacConkey agar plate.

### 2.3. Susceptibility Testing

Susceptibility of* Salmonella* isolates against antimicrobials was detected using agar dilution method according to the guidelines of the Clinical and Laboratory Standards Institute (CLSI). Minimal inhibitory concentrations (MICs) for each antibiotic were determined by VITEK 2 compact (bioMérieux, Marcy-l'Etoile, France). Susceptibility interpretations were based on CLSI M100-S23 clinical breakpoints [10]. The antibiotics used included carbapenems (imipenem and meropenem), penicillins (ampicillin and amoxicillin clavulanate), cephalosporins (ceftriaxone and ceftazidime), fluoroquinolones (ciprofloxacin and levofloxacin), trimethoprim-sulfamethoxazole, and aminoglycosides (amikacin and gentamicin). Results were included in the analysis only when the corresponding QC isolates tested were within the acceptable range according to CLSI guidelines. Chloramphenicol susceptibility for* S. *Typhi and* S.* Paratyphi was not tested according to the policy of the Indonesian Microbiology Association.

### 2.4. Statistics

The data were analyzed statistically using Statistical Package for Social Sciences (SPSS, version 24.0.0.0). Categorical data were presented as frequency (percentage), while numerical data as mean ± SD or median (range).

### 2.5. Ethical Considerations

The study protocol was approved by the Ethical Committee of Siloam Hospital, Karawaci, Tangerang, Banten, Indonesia.

## 3. Results

### 3.1. Distribution of Isolates

A total of 168* Salmonella enterica *serovar Typhi and Paratyphi isolates were collected in the study period, including 93 (55.4%)* S.* Typhi and 75 (44.6%)* S.* Paratyphi* A*. There were male preponderance over female (100 (59.5%) versus 68 (40.5%)). The median age was 19 years (range 1 to 80 years). Most of the isolates, 156 (92.9%), were from children aged 6–18 years and adult population, 8 (4.8%) were from children below 5 years of age, and 4 (2.4%) were from adult above 60 years of age. There was no mortality. Demographic characteristics of* Salmonella* isolates were shown in Table 1.

### 3.2. Antimicrobial Susceptibility

*Salmonella* Typhi in this study showed low resistance against most antimicrobials such as ampicillin, trimethoprim-sulfamethoxazole, ceftriaxone, ciprofloxacin, and levofloxacin (5.4%, 8.6%, 0.0%, 1.1%, and 3.2%, resp.). There were also low resistance against the carbapenems (meropenem and imipenem). A decrease of resistance rates against ampicillin, amoxicillin clavulanate, cefotaxime, trimethoprim-sulfamethoxazole, and ampicillin and trimethoprim-sulfamethoxazole was observed between 2011 to 2015. Other antimicrobials showed stable resistance rates. The resistance pattern of* S. *Typhi is shown in Table 2. The minimum inhibitory concentration (MIC) to antimicrobials of* Salmonella* Typhi isolates is shown in Table 3.

*Salmonella* Paratyphi* A* in this study showed low resistance against most antimicrobials such as ampicillin, trimethoprim-sulfamethoxazole, ceftriaxone, cefotaxime, ciprofloxacin, and levofloxacin (6.7%, 8.0%, 1.3%, 2.7%, 0.0%, and 1.3%, resp.). Similarly, there was no resistance against the carbapenems. A decrease of resistance rates against trimethoprim-sulfamethoxazole was observed. Other antimicrobials showed stable resistance rates. The resistance pattern of* S. *Paratyphi* A* is shown in Table 4. The minimum inhibitory concentration (MIC) to antimicrobials of* Salmonella* Paratyphi* A *isolates is shown in Table 5.

## 4. Discussion

The* Salmonella *serovars found in this study were 55.4%* S.* Typhi and 44.6%* S.* Paratyphi* A*, which was in line with a study in Indonesia that showed the predominance of* S.* Typhi over* S.* Paratyphi [11]. Studies in India and China stated that the reason for increasing prevalence of* S. *Paratyphi* A* over* S. *Typhi in recent years was the increased use of Vi polysaccharide vaccine [12]. Even though, in this study, there were no data on the patients' history of vaccination, the data of this study showed that salmonellosis affected mostly young and middle-aged population as 92.9% of these populations have more contact with water and food that were contaminated with* Salmonella *[13]. The increased public standard of living resulted the reduction in the frequency of* Salmonella *isolation in a study in China, due to the reduced consumption of contaminated water and food [14].

The antimicrobial resistance rates of* S.* Typhi differ among countries in the world. A study in Pakistan showed that resistance rates of of* S.* Typhi and Paratyphi were 88.2% for fluoroquinolone, 66.1% for ampicillin, and 66.5% for trimethoprim-sulfamethoxazole [15]. Another study in China showed that resistance rates of* S.* Typhi and Paratyphi were 13.5% and 5.9% for ciprofloxacin, 5.4% and 0.8% for levofloxacin, 5.4% and 1.4% for sulfamethoxazole, 10.8% and 2.0% for ampicillin, 5.4% and 0.0% for ceftriaxone, and 0.0% for meropenem and imipenem [14]. A study in Nepal showed 1.8% resistance of* S.* Typhi against ampicillin and 3.9% of* S.* Paratyphi against ciprofloxacin, while there was no resistance against trimethoprim-sulfamethoxazole [16]. A study in Asian countries showed that resistance rates in Bangladesh were 39.5% for ciprofloxacin, 68.4% for ampicillin, 57.9% for trimethoprim, and 68.4% for sulfamethoxazole, in Vietnam 0.0% for ciprofloxacin, and 80.4% for ampicillin, trimethoprim, and sulfamethoxazole, while in Indonesia they were 0.0% for ciprofloxacin, 1.8% for ampicillin and trimethoprim, and 3.6% for sulfamethoxazole [17]. A community-based study from 2001 to 2003 in Indonesia showed low resistance rates of* S.* Typhi and* S.* Paratyphi, where only 2.5% isolates of* S.* Typhi were resistant against ampicillin, while there was no resistance against trimethoprim-sulfamethoxazole, ceftriaxone, or ciprofloxacin [11]. A hospital-based study from 2006 to 2010 in Indonesia also found similar results;* S.* Typhi showed no resistance against trimethoprim-sulfamethoxazole, ciprofloxacin, and meropenem, 0.9% against ceftriaxone, 1.6% against cefotaxime, and 1.9% against ampicillin [18]. This study also found similar results to studies in Indonesia; the low resistance of* S.* Typhi and* S.* Paratyphi isolates from 2011 to 2015 against most antimicrobials such as ampicillin, trimethoprim-sulfamethoxazole, ceftriaxone, ciprofloxacin, and levofloxacin (5.4%, 8.6%, 0.0%, 1.1%, 3.2% and 6.7%, 8.0%, 1.3%, 2.7%, 0.0%, and 1.3%, resp.). In this study, the frequency of MDR* S.* Typhi would be no more than 4.3%. The resistance rate of* S. *Typhi isolates against ampicillin and trimethoprim-sulfamethoxazole was only 4.3%, so even if all the isolates were resistant to chloramphenicol, the MDR rate would not be higher than that. A study stated that there were differences in the resistance rates between* S. *Paratyphi* A* or* S. *Typhi; therefore they have to be considered as different diseases [19]. This study, on the contrary, indicated no significant different resistance rates between* S. *Typhi and* S. *Paratyphi* A*.

The two antimicrobial resistance mechanisms in* S. *Typhi were the plasmid-mediated mechanism and the chromosomal-mediated mechanism [20]. Plasmids that transfer the antimicrobial resistance in* S. *Typhi are the plasmids of incompatibility group (Inc) HI1. The first IncHI1 plasmid was isolated in Mexico City in 1972 and rendered resistance to chloramphenicol, tetracycline, streptomycin, and sulphonamides. Afterwards, MDR* S. *Typhi associated with IncHI1 plasmids were found worldwide. The chromosomal-mediated drug resistance against fluoroquinolones was the result of selective selection on the bacterial population by the usage of the antimicrobial. The single point mutation in the quinolone resistance determining region (QRDR) of the topoisomerase gene* gyrA* that encodes DNA gyrase was the resistance gene against fluoroquinolones. Other resistance genes of* S. *Typhi were* cat* for chloramphenicol,* tetA* for tetracycline,* bla* for ampicillin,* strA-strB* for streptomycin, and* dfrA* and* sul1* for trimethoprim and sulfonamide [20]. A study found very closely related plasmids that have a common backbone carrying identical resistance insertions and were present in* S. *Typhi and* S. *Paratyphi* A*. This study determined the DNA sequence of an IncHI1 plasmid, pAKU_1, encoding MDR in a* S. *Paratyphi* A*. It strongly suggests that there had been transfer of a plasmid between* S. *Typhi and* S. *Paratyphi* A* [21].

The explanation of different resistance pattern in the world was that there are different strains of* S.* Typhi and* S.* Paratyphi in different regions which also have different genes that contributed to resistance against antimicrobials [17]. A study in 2008 on haplotypes and pathotypes of* S. *Typhi in Jakarta, Indonesia, found 9 serovar strains of haplotypes which were H1, H8, H42, H45, H50, H52, H59, H84, and H85. Haplotypes H59 and H8 were dominating the isolates, 53% and 24%, respectively. Haplotypes of H42, H50, H59, and H85 were also found in previous studies, whereas H85 had been isolated in 1987 and 2003. Haplotype H59 which is associated with j and z66 flagellum expression seemed to be the specific phenotype in Indonesia, as it has been consistently isolated over a 30-year period. A study on the DNA profiles of* S.* Typhi in Indonesia showed clear differences according to regions but all the* S. *Typhi isolates showed similar phenotypes which were susceptible to chloramphenicol, ampicillin, and trimethoprim-sulfamethoxazole [22]. The MDR rate in this study was 4.3%, which confirmed the result of previous studies that MDR rate in Indonesia is low. Only one strain of H58 that was resistant to fluoroquinolone was found from a French traveler returning from Indonesia in 2003. Mutations in DNA gyrase were not found in the study, which indicates that there was no recent clonal expansion of H58 in Indonesia. Studies found that MDR* S*. Typhi strains belong to haplotype H58, which spread widely over the Indochina Peninsula, the Indian subcontinent, and Africa but is not prevalent in Indonesia [23–28]. It could be concluded that there is a distinct epidemiological dynamic of the enteric fever in Indonesia. As stated above, there was evidence of transfer of a plasmid responsible for multidrug resistance between* S. *Typhi and* S. *Paratyphi* A *[21]. If the haplotype H58* S. *Typhi is not found in Indonesia, the IncHI1 plasmid that could transfer multidrug resistance to* S. *Paratyphi would also be absent. This would explain the nonsignificant difference of resistance rates between* S. *Typhi and* S. *Paratyphi* A *in this study.

According to the 2006 Indonesian Typhoid Fever Disease Control Guidelines, chloramphenicol, ampicillin, or trimethoprim-sulfamethoxazole is used as first-line antimicrobials, and if there is resistance to the first-line antimicrobials then the second line antimicrobials; ceftriaxone, cefixime, or quinolones is indicated [29]. In Indonesia, a study on hospitalized adult typhoid patients found that ceftriaxone was the antimicrobial most commonly used followed by levofloxacin, ciprofloxacin, meropenem, metronidazole, and ampicillin-sulbactam [30]. The frequent use of second line antimicrobials for enteric fever in Indonesia could also be the explanation for the low MDR and low resistance rates for first-line antimicrobials in this study, where a study in Nepal found that the discontinuation of chloramphenicol and trimethoprim-sulfamethoxazole for long periods might be the cause of reemergence of sensitive* S.* Typhi and* S.* Paratyphi [16].

There were limitations of this study that should be mentioned. The samples of this study were blood collected from admitted patients in a hospital; thus the antimicrobial resistance pattern was restricted to the patients of the hospital. The resistance pattern must be interpreted cautiously, as isolates might not represent the entire population of Banten province or Indonesia. Another issue was the unavailability of data on chloramphenicol susceptibility because of the policy of the Indonesian Microbiology Association not to test chloramphenicol for* S. *Typhi and* S.* Paratyphi anymore. Other antimicrobial not tested was nalidixic acid. In vitro nalidixic acid is more appropriate to test for in vivo fluoroquinolone resistance.

## 5. Conclusions

The study discovered that in the 2011–2015 period, the resistance rates against most antimicrobials and even MDR rate of* S. *Typhi and* S.* Paratyphi were low, in accordance with previous studies in different regions of Indonesia in the 2001–2003, 2006–2010, and 2008 periods. It emphasizes that there is a distinct epidemiological dynamic of the enteric fever in Indonesia. To prevent the occurrence of resistance and MDR* S. *Typhi, it is imperative to maintain continuous monitoring of antimicrobial resistance and follow a rational prescription of antimicrobials based on the local antimicrobial pattern.

## Figures and Tables

**Table 1:** Demographic characteristics of *Salmonella* isolates in Siloam Hospital, Karawaci, Tangerang, Banten, Indonesia.

	*n*	%
Serovars		
*Salmonella enterica* serovar Typhi	93	55.4
*Salmonella enterica* serovar Paratyphi* A*	75	44.6
Sex		
Male	68	40.5
Female	100	59.5
Age group		
≤5 years	8	4.8
6–18 years	68	40.5
19–59 years	88	52.4
≥60 years	4	2.4

**Table 2:** Antimicrobial resistance pattern of *Salmonella *Typhiisolates in Siloam Hospital, Karawaci, Tangerang, Banten, Indonesia.

Antimicrobial	2011 (*n* = 14)		2012 (*n* = 16)		2013 (*n* = 17)		2014 (*n* = 27)		2015 (*n* = 19)		Total (*n* = 93)	
	*n*	%	*n*	%	*n*	%	*n*	%	*n*	%	*n*	%
AMP	2	14.3	2	12.5	1	5.9	0	0.0	0	0.0	5	5.4
AMC	1	7.2	1	6.3	1	5.9	0	0.0	0	0.0	3	3.2
CRO	0	0.0	0	0.0	0	0.0	0	0.0	0	0.0	0	0.0
CT	2	14.3	1	6.3	0	0.0	0	0.0	0	0.0	3	3.2
MER	0	0.0	0	0.0	0	0.0	1	3.7	0	0.0	1	1.1
IPM	0	0.0	0	0.0	0	0.0	1	3.7	0	0.0	1	1.1
TMP-SMX	5	35.7	1	6.3	1	5.9	1	3.7	0	0.0	8	8.6
AMP + TMP-SMX	2	14.3	1	6.3	1	5.9	0	0.0	0	0.0	4	4.3
CIP	0	0.0	1	6.3	0	0.0	0	0.0	0	0.0	1	1.1
LEVX	0	0.0	1	6.3	1	5.9	1	3.7	0	0.0	3	3.2

AMP, ampicillin; AMC, amoxicillin clavulanate; CRO, ceftriaxone; CT, cefotaxime; MER, meropenem; IPM, imipenem; TMP-SMX, trimethoprim-sulfamethoxazole; AKN, amikacin; GEN, gentamicin; CIP, ciprofloxacin; LEVX, levofloxacin.

**Table 3:** Minimum inhibitory concentrations to antimicrobials of *Salmonella *Typhiisolates in Siloam Hospital, Karawaci, Tangerang, Banten, Indonesia.

Antimicrobial	MIC (*μ*g/mL)	2011		2012		2013		2014		2015		Total	
		(*n* = 14)		(*n* = 16)		(*n* = 17)		(*n* = 27)		(*n* = 19)		(*n* = 93)	
		*n*	%	*n*	%	*n*	%	*n*	%	*n*	%	*n*	%
AMP	≤2	11	78,6	10	62,5	11	64,7	20	74,1	16	84,2	68	73,1
	4	1	7,1	4	25,0	3	17,6	4	14,8	2	10,5	14	15,1
	8	0	0,0	0	0,0	2	11,8	3	11,1	1	5,3	6	6,5
	≥32	2	14,3	2	12,5	1	5,9	0	0,0	0	0,0	5	5,4

AMC	≤2	13	92,9	15	93,8	16	94,1	27	100,0	19	100,0	90	96,8
	4	1	7,1	1	6,3	1	5,9	0	0,0	0	0,0	3	3,2

CRO	≤1	14	100,0	16	100,0	17	100,0	27	100,0	19	100,0	93	100,0
	>1	0	0,0	0	0,0	0	0,0	0	0,0	0	0,0	0	0,0

CT	≤1	12	85,7	15	93,8	17	100,0	27	100,0	19	100,0	90	96,8
	>1	2	14,3	1	6,3	0	0,0	0	0,0	0	0,0	3	3,2

MER	≤0.25	14	100,0	16	100,0	17	100,0	26	96,3	19	100,0	92	98,9
	>0.25	0	0,0	0	0,0	0	0,0	1	3,7	0	0,0	1	1,1

IPM	≤1	14	100,0	15	93,8	17	100,0	26	96,3	19	100,0	91	97,8
	2	0	0,0	1	6,3	0	0,0	0	0,0	0	0,0	1	1,1
	>2	0	0,0	0	0,0	0	0,0	1	3,7	0	0,0	1	1,1

TMP-SMX	≤20	9	64,3	15	93,8	16	94,1	26	96,3	19	100,0	85	91,4
	≥320	5	35,7	1	6,3	1	5,9	1	3,7	0	0,0	8	8,6

CIP	≤0.25	12	85,7	15	93,8	12	70,6	21	77,8	19	100,0	79	84,9
	0,5	0	0,0	0	0,0	1	5,9	0	0,0	0	0,0	1	1,1
	1	2	14,3	0	0,0	4	23,5	6	22,2	0	0,0	12	12,9
	4	0	0,0	1	6,3	0	0,0	0	0,0	0	0,0	1	1,1

LEVX	≤0.12	14	100,0	14	87,5	13	76,5	19	70,4	19	100,0	79	84,9
	0,25	0	0,0	0	0,0	1	5,9	0	0,0	0	0,0	1	1,1
	0,5	0	0,0	1	6,3	2	11,8	1	3,7	0	0,0	4	4,3
	1	0	0,0	0	0,0	0	0,0	6	22,2	0	0,0	6	6,5
	2	0	0,0	0	0,0	0	0,0	0	0,0	0	0,0	0	0,0
	≥8	0	0,0	1	6,3	1	5,9	1	3,7	0	0,0	3	3,2

MIC, minimum inhibitory concentrations; AMP, ampicillin; AMC, amoxicillin clavulanate; CRO, ceftriaxone; CT, cefotaxime; MER, meropenem; IPM, imipenem; TMP-SMX, trimethoprim-sulfamethoxazole; AKN, amikacin; GEN, gentamicin; CIP, ciprofloxacin; LEVX, levofloxacin.

**Table 4:** Antimicrobial resistance pattern of *Salmonella *Paratyphi* A* isolates in Siloam Hospital, Karawaci, Tangerang, Banten, Indonesia.

Antimicrobial	2011		2012		2013		2014		2015		Total	
	(*n* = 14)		(*n* = 22)		(*n* = 17)		(*n* = 18)		(*n* = 4)		(*n* = 75)	
	*n*	%	*n*	%	*n*	%	*n*	%	*n*	%	*n*	%
AMP	1	7.1	2	9.1	1	5.9	1	5.6	0	0.0	5	6.7
AMC	1	7.1	0	0.0	1	5.9	1	5.6	0	0.0	3	4.0
CRO	0	0.0	1	4.5	0	0.0	0	0.0	0	0.0	1	1.3
CT	1	7.1	1	4.5	0	0.0	0	0.0	0	0.0	2	2.7
MER	0	0.0	0	0.0	0	0.0	0	0.0	0	0.0	0	0.0
IPM	0	0.0	0	0.0	0	0.0	0	0.0	0	0.0	0	0.0
TMP-SMX	5	35.7	0	0.0	0	0.0	1	5.6	0	0.0	6	8.0
AMO + TMP-SMX	1	7.1	0	0.0	0	0.0	0	0.0	0	0.0	1	1.3
CIP	0	0.0	0	0.0	0	0.0	0	0.0	0	0.0	0	0.0
LEVX	0	0.0	0	0.0	0	0.0	1	5.6	0	0.0	1	1.3

AMP, ampicillin; AMC, amoxicillin clavulanate; CRO, ceftriaxone; CT, cefotaxime; MER, meropenem; IPM, imipenem; TMP-SMX, trimethoprim-sulfamethoxazole; AKN, amikacin; GEN, gentamicin; CIP, ciprofloxacin; LEVX, levofloxacin.

**Table 5:** Minimum inhibitory concentrations to antimicrobials of *Salmonella* Paratyphi* A *isolates in Siloam Hospital, Karawaci, Tangerang, Banten, Indonesia.

Antimicrobial	MIC (*μ*g/mL)	2011		2012		2013		2014		2015		Total	
		(*n* = 14)		(*n* = 22)		(*n* = 17)		(*n* = 18)		(*n* = 4)		(*n* = 75)	
		*n*	%	*n*	%	*n*	%	*n*	%	*n*	%	*n*	%
AMP	≤2	13	92,9	18	81,8	13	76,5	13	72,2	2	50,0	59	78,7
	4	0	0,0	2	9,1	1	5,9	1	5,6	1	25,0	5	6,7
	8	0	0,0	0	0,0	2	11,8	3	16,7	1	25,0	6	8,0
	≥32	1	7,1	2	9,1	1	5,9	1	5,6	0	0,0	5	6,7

AMC	≤2	13	92,9	22	100,0	16	94,1	17	94,4	4	100,0	72	96,0
	4	1	7,1	0	0,0	1	5,9	1	5,6	0	0,0	3	4,0

CRO	≤1	14	100,0	21	95,5	17	100,0	18	100,0	4	100,0	74	98,7
	>1	0	0,0	1	4,5	0	0,0	0	0,0	0	0,0	1	1,3

CT	≤1	13	92,9	21	95,5	17	100,0	18	100,0	4	100,0	73	97,3
	>1	1	7,1	1	4,5	0	0,0	0	0,0	0	0,0	2	2,7

MER	≤0.25	14	100,0	22	100,0	17	100,0	18	100,0	4	100,0	75	100,0
	>0.25	0	0,0	0	0,0	0	0,0	0	0,0	0	0,0	0	0,0

IPM	≤1	14	100,0	21	95,5	17	100,0	18	100,0	4	100,0	74	98,7
	2	0	0,0	1	4,5	0	0,0	0	0,0	0	0,0	1	1,3
	>2	0	0,0	0	0,0	0	0,0	0	0,0	0	0,0	0	0,0

TMP-SMX	≤20	9	64,3	22	100,0	17	100,0	17	94,4	4	100,0	69	92,0
	≥320	5	35,7	0	0,0	0	0,0	1	5,6	0	0,0	6	8,0

CIP	≤0.25	13	92,9	21	95,5	14	82,4	13	72,2	3	75,0	64	85,3
	0,5	0	0,0	0	0,0	0	0,0	0	0,0	0	0,0	0	0,0
	1	1	7,1	1	4,5	3	17,6	5	27,8	1	25,0	11	14,7
	4	0	0,0	0	0,0	0	0,0	0	0,0	0	0,0	0	0,0

LEVX	≤0.12	14	100,0	21	95,5	15	88,2	10	55,6	2	50,0	62	82,7
	0,25	0	0,0	0	0,0	0	0,0	1	5,6	0	0,0	1	1,3
	0,5	0	0,0	1	4,5	2	11,8	2	11,1	2	50,0	7	9,3
	1	0	0,0	0	0,0	0	0,0	4	22,2	0	0,0	4	5,3
	2	0	0,0	0	0,0	0	0,0	0	0,0	0	0,0	0	0,0
	≥8	0	0,0	0	0,0	0	0,0	1	5,6	0	0,0	1	1,3

MIC, minimum inhibitory concentrations; AMP, ampicillin; AMC, amoxicillin clavulanate; CRO, ceftriaxone; CT, cefotaxime; MER, meropenem; IPM, imipenem; TMP-SMX, trimethoprim-sulfamethoxazole; AKN, amikacin; GEN, gentamicin; CIP, ciprofloxacin; LEVX, levofloxacin.
